# Right-Sided Cardiac Thrombosis and Pulmonary Thromboembolism in Chronic Chagas Disease: A Review of Clinical Features and Post-Mortem Examination

**DOI:** 10.31083/j.rcm2506220

**Published:** 2024-06-19

**Authors:** Reinaldo B. Bestetti, Marcelo José Ferreira Soares, Rosemary Furlan-Daniel, Augusto Cardinalli-Neto, Marcelo A. Nakazone

**Affiliations:** ^1^Department of Medicine, University of Ribeirão Preto, 14096-900 Ribeirão Preto, Brazil; ^2^Postgraduate Division of São José do Rio Preto Medical School, 15090-000 São José do Rio Preto, Brazil; ^3^Department of Cardiology and Cardiovascular Surgery, São José do Rio Preto Medical School, 15090-000 São José do Rio Preto, Brazil

**Keywords:** pulmonary embolism, Chagas disease, Chagas cardiomyopathy, chronic heart failure, cardiac thrombus, trypanosoma cruzi

## Abstract

Pulmonary thromboembolism (PE) is a potential major complication in patients with chronic Chagas heart disease (CChD). The source of PE is the right-sided chambers instead of deep vein thrombosis. Little is known regarding risk factors, clinical picture, and the clinical course of patients with PE secondary to CChD. The aim of this review was to try to provide doctors with such data. We searched for papers related to PE in CChD patients in the PUBMED from 1955 to 2020. Twenty-six manuscripts were retrieved, of which 12 fulfilled entry criteria and were included in the study. Right-sided cardiac thrombosis or PE was confirmed on morphological or imaging studies. A total of 431 patients with PE were reported. Age varied from 30 to 85 years. About 332 patients were reported to have chronic heart failure (CHF), whereas 41 (9%) sudden cardiac death (SCD) at autopsy. Clinical manifestations reported were sudden onset dyspnea was found in 1 patient, haemoptysis in 2, worsening CHF in 2, and chest pain in 1. An X-ray chest was reported for 6 patients: abnormalities consistent with PE were found in 3. The resting electrocardiogram (ECG) was reported for 5 patients: it was abnormal in all. One study reported a mean left ventricular ejection fraction of 42.1 ± 18.7%. The prevalence of right-sided cardiac thrombosis varied from 66% to 85% patients. PE was the cause of death in 17% of patients. The clinical diagnosis of PE in patients with Chagas cardiomyopathy (ChCM) is very difficult in the absence of a prediction score that performs well. However, in the presence of haemoptysis or worsening heart failure (HF), abnormal ECG, or chest X-ray, the diagnosis of PE should be raised, and patients promptly referred to detailed Doppler Tissue Echocardiography and computed tomography angiography, and treated in a timely manner.

## 1. Introduction

Chronic Chagas disease still is a major health problem in Latin America, and now it has spread throughout the world because of immigration. The disease is caused by the protozoan *Trypanosoma cruzi*, which is transmitted to humans through the faeces of a sucking bug. Chagas cardiomyopathy (ChCM) is the most significant clinical manifestation of chronic Chagas disease and appears up to two decades after initial infection [1].

ChCM manifests as chronic heart failure with reduced left ventricular ejection fraction (HFREF), sudden cardiac death [1], atrioventricular (AV) blocks [2], malignant ventricular arrhythmias [3], myocardial infarction/ischemia with normal epicardial coronary arteries [4], and thromboembolism [5]. HFREF is the main clinical manifestation of ChCM [6], its prognosis is worse than that observed in patients with non-Chagas disease heart failure [7]. Heart transplantation is the treatment of choice in terminal stages [8], and pulmonary thromboembolism (PE) is a common complication [5].

The prevalence of PE in patients with ChCM without overt HFREF is about 0.6% [9]. However, PE can be observed in up to 37% of patients with HFREF secondary to ChCM [10], which seems to be higher than that observed in non-Chagas disease patients. In fact, the prevalence of PE in the general population is about 60/100,000 inhabitants, and its incidence approaches 1/1000 people annually. The prevalence of deep vein thrombosis, the major cause of PE in non-Chagas disease patients, is around 124/100,000 inhabitants [11].

The diagnosis of PE is challenging in non-Chagas disease patients because the clinical presentation is non-specific; consequently, the diagnosis of this condition may be made in only about 10% of cases [12]. Scores have been used to make the diagnosis of PE in non-Chagas disease patients [13, 14, 15, 16]. However, it is very difficult to extrapolate such scores to patients with ChCM because the main cause of PE is right-sided thrombus, and not deep vein thrombosis [5].

Since little is known regarding clinical presentation and risk factors for patients with PE secondary to ChCM, and the prognosis of PE in patients with this condition can be dismal, we wrote this review in an attempt to provide doctors with the best evidence possible to manage this potentially lethal disorder.

## 2. Search Strategy

We have searched for papers related to PE in ChCM patients using PUBMED since the beginning of the MEDLINE database. The time-span for the search of potential papers was from 1955 to 2020. We used the terms “Chagas disease and pulmonary embolism” as well as “Chagas disease and cardiac thrombosis”, which were the main line of research. Additionally, we used the terms “Chagas cardiomyopathy and pulmonary embolism”, or “chronic Chagas disease and pulmonary embolism” as secondary lines of research to find manuscripts related to PE in the setting of chronic Chagas disease. A search of the references included in the retrieved papers was also performed. We included all papers reporting clinical aspects and morphological findings related to either PE or cardiac thrombosis in the setting of chronic Chagas disease irrespective of age, cross-sectional or longitudinal cohort studies design, or case reports; papers reporting only morphological features were also included in the study. In all circumstances, right-sided cardiac thrombosis or PE were diagnosed by post-mortem examination or imaging studies. We ruled out review papers and papers reporting the association of chronic Chagas disease-induced PE with other concomitant diseases.

In total, 26 manuscripts were retrieved. All were read by abstract; four manuscripts did not report on Chagas disease and were ruled out from the investigation. The remaining 22 papers were read in full. Of these, three papers were an overview of Chagas disease, five papers related to chronic Chagas disease with no PE, one paper reported on the association between Chagas disease and schistosomiasis, and one paper was focused on Chagas disease’s association with obstructive coronary artery disease. These were all also excluded from the work. The remaining 12 manuscripts were included in the study. Because of the small number of patients that could be included in the investigation, a formal meta-analysis could not be carried out.

## 3. Clinical, Radiological, Electrocardiographic, and Echocardiographic Aspects 

A total of 431 patients with Chagas cardiomyopathy were reported to have PE. However, a clinicopathological correlation was only done occasionally. Age varied between 30 and 85 years. PE was clinically manifested by sudden onset dyspnea in one patient [17], haemoptysis in two papers [17, 18], and worsening chronic heart failure (CHF) in two others [19, 20]. One patient had chest pain associated with PE [18]. 41 patients had an association with sudden cardiac death (SCD) at autopsy, but whether the PE or a malignant arrhythmia was the cause of death could not be established in that study [5]. All other 332 (77%) reported Chagas disease patients had a clinical picture consistent with CHF; however, the diagnosis of PE was not apparently suspected in most patients [21, 22, 23].

PE was suspected because an abnormal chest X-ray in three cases [17, 18, 19]; in one case, a chest computed tomography showed bilateral aneurysms of pulmonary arteries, superior vena cava thrombosis, and PE at pulmonary angiography [18]. Cardiomegaly without evidence of thromboembolism was reported in two other cases [21], and bilateral perihilar opacities in another patient [18].

**Fig. 1.**
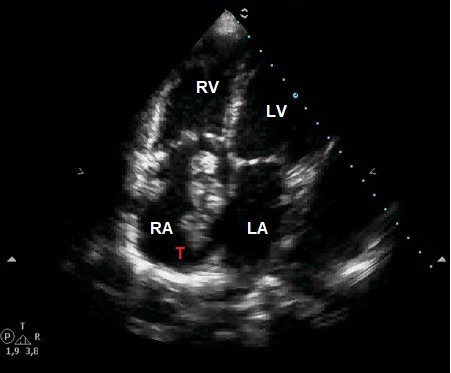
**A huge thrombus is seen inside the right atrium.** T, thrombus; RA, right atrium; LA, left atrium; RV, right ventricle; LV, left ventricle. Reproduced with permission from Acta Cardiol 2011; 66: 67–69 [25].

**Fig. 2.**
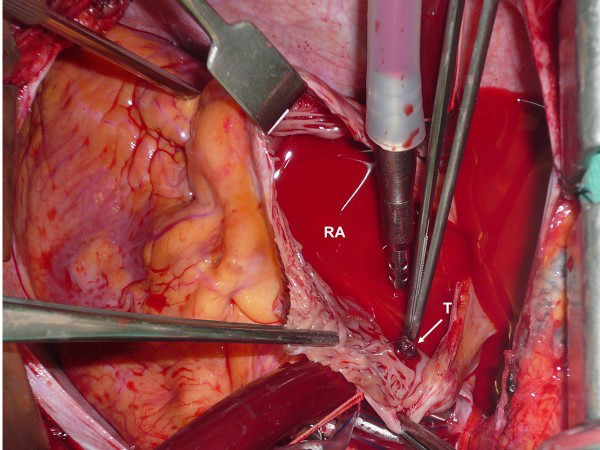
**A thrombus can be seen inside the right atrium during cardiac operation from the same patient shown in Fig. 1.** T, thrombus; RA, right atrium.

An abnormal resting electrocardiogram (ECG) was observed in four patients associated with PE: atrial fibrillation was seen in two patients [22], right bundle branch block in four [17, 18, 20, 22], left anterior fascicular block in three [18, 20, 22], left atrial enlargement in three [18, 21], and pathological Q waves in two [21, 22]. Detailed echocardiography for each patient was not reported. In one study, the mean left ventricular ejection fraction (LVEF) of all patients with CHF included in the investigation was 42.1 ± 18.7% [24]. In another study, an asymptomatic patient with right bundle branch block in the resting ECG, normal chest X-ray, right ventricular dilatation, right ventricular hypokinesia, and right atrial enlargement at transthoracic echocardiogram was also found to have a massive right atrium thrombus [25] with no episode of PE. Fig. 1 (Ref. [25]) and Fig. 2 show the thrombus during the cardiac operation.

## 4. Post-Mortem Examination

PE was detected in about 17% to 55% of autopsied patients [5, 10, 21, 23]. At autopsy, right-sided cardiac thrombosis was found in 151 out of 227 (66%) patients with PE in the study by Oliveira *et al*. [5], and in 35 of 41 (85%) patients in the study by Arteaga-Fernández *et al*. [10]. In patients with CHF with PE, 57 of 186 (30%) patients had no right-sided cardiac thrombosis in the study by Oliveira *et al*. [5], and 6 of 41 (14%) patients in the study reported by Arteaga-Fernández *et al*. [10]. In chronic Chagas disease patients without HFREF, right-sided cardiac thrombosis was found in 18 of 29 (62%) patients in the study by Anselmi *et al*. [26].

PE was the cause of death in 17% of patients with CHF with right-sided cardiac thrombosis, and in 5% of those with CHF but no right-sided cardiac thrombosis in the study by Oliveira *et al*. [5]. PE was found in 41 of 227 (18%) patients with PE who experienced SCD, but no causal relationship could be confirmed between the conditions. In one patient, a cardiac thrombus was firmly attached to the lead of a pacemaker implanted in the right ventricle, which was considered the cause of the patient’s poor prognosis [20]. Table 1 (Ref. [5, 10, 17, 18, 19, 20, 21, 22, 23, 24, 25, 26]) summarizes the papers included in this study.

**Table 1.** **Clinical characteristics and post-mortem examination findings associated with pulmonary embolism or isolated right-sided cardiac thrombosis in patients with chronic Chagas heart disease**.

Author	Year	Clinical Aspects	ECG	Chest X-Ray	Postmortem examination
Anselmi *et al*. [26]	1966	-	-	-	RSCT: 79%
Castagnino *et al*. [17]	1978	Sudden Onset of Dyspnea; Haemoptysis	PQ wave; RBBB	Suggestive of PE	-
Oliveira *et al*. [5]	1983	CHF	-	-	PE: 18%
Corrêa de Araujo *et al*. [21]	1985	CHF	ST-T changes, LAE, LVH, VPC, PQ wave	Cardiomegaly	PE: 10%
Hurtado Santos *et al*. [19]	1985	Worsening CHF	-	Suggestive of PE	-
Bestetti *et al*. [22]	1987	CHF	AF, RBBB, LAFB	-	PE: 12%
Arteaga-Fernández *et al*. [10]	1989	CHF	-	-	PE: 37%
Bittencourt *et al*. [23]	1990	CHF	VPC, LVH, LAFB, IVB	-	PE: 55%
Pereira de Godoy *et al*. [18]	2000	Haemoptysis, chest pain	RBBB, LAFB	Suggestive of PE	-
Medeiros *et al*. [20]	2001	Worsening CHF	RBBB, LAFB	-	-
**Bestetti *et al*. [25]	2011	No cardiac complain	RBBB	Normal	-
de Macedo *et al*. [24]	2018	CHF	-	-	PE: 21%

RSCT, right-sided cardiac thrombus; PE, pulmonary embolism; CHF, chronic heart failure; PQ, pathological; RBBB, right bundle branch block; LAE, left atrial enlargement; LVH, left ventricular hypertrophy; VPC, ventricular premature contractions; AF, atrial fibrillation; LAFB, left anterior fascicular block; IVB, intraventricular block; ECG, electrocardiogram. **Right atrial thrombus at transthoracic echocardiogram.

## 5. Discussion

This review suggests that haemoptysis and worsening heart failure (HF) may be associated with PE in patients with ChCM; conversely, syncope and chest pain are not associated with PE in patients with PE and ChCM. The vast majority of cases of PE in patients with this condition were associated with HF. In addition, a normal ECG or chest X-ray have not been observed in patients with PE secondary to ChCM as well. About one quarter of autopsied reported patients with chronic Chagas disease presented underlying PE, which was the cause of death in up to 17% of them. Moreover, this investigation also shows that PE was associated with right-sided cardiac thrombosis in patients with this condition, and not with deep vein thrombosis, as commonly observed in non-Chagas disease patients with PE [12]. Collectively, these findings can help doctors working in rural areas to raise the suspicion of PE in patients with ChCM, thus referring patients to a referral centre for a detailed Doppler echocardiography examination, computed tomography angiography, and prompt treatment in the case of the diagnosis of PE in patients with ChCM.

The diagnosis of PE is rare in a general population comprised of non-Chagas disease patients with either dyspnea or chest pain at the emergency department [27, 28]. Our study appears to confirm that in Chagas disease, patients with dyspnea did not necessarily develop PE. However, the situation might be different in patients with worsening dyspnea. In our study, patients were reported to have worsening dyspnea, a condition that increases the probability of the diagnosis of PE in non-Chagas disease patients [29], and may have the same impact on ChCM patients. Given the potential association of PE with HFrEF in patients with ChCM, we suggest that in every patient with no clear reason for worsening dyspnea, the diagnostic possibility of PE should be raised.

Chest pain has not been associated with PE in non-Chagas disease patients [27, 28]. Chest pain was reported in only one patient with PE secondary to ChCM. Therefore, isolated chest pain appears not to herald PE in patients with ChCM as well. By contrast, the presence of haemoptysis is associated with an 8% probability of PE in non-Chagas disease patients [15]. Thus, ChCM patients presenting with haemoptysis may also have a high probability of experiencing PE.

The incidence of unexplained syncope associated with PE in non-Chagas disease patients at 30 days of presentation at an emergency department may be as low as 0.6%, and mortality associated with PE still lower (0.04%) [30]. Notably, unexplained syncope was not reported in patients with PE with ChCM. SCD in patients with chronic Chagas disease is caused by malignant arrhythmia in the overwhelming majority of cases [31]. Therefore, this review is consistent with the notion that doctors working on the frontline should consider malignant arrhythmia instead of PE in patients with syncope associated with ChCM.

Abnormal Chest X-rays were only found in 12% of patients with PE in non-Chagas disease [32, 33]. In our review, however, patients with ChCM have been reported to have abnormal chest X-ray associated with PE. It seems that a simple chest X-ray may be of value, especially in the setting of high clinical suspicion, to rule out the diagnosis of PE in patients with ChCM.

A resting ECG is poorly associated with PE in non-Chagas disease patients. The pattern of right ventricular strain seems to be the most frequent abnormality in the resting ECG of non-Chagas disease patients associated with PE (11.1%) in comparison with controls [34]. This ECG abnormality has not been reported in patients with PE secondary to ChCM. However, all patients with PE associated with ChCM had abnormal an ECG. Therefore, our review suggests that a normal ECG probably discards the diagnosis of PE in patients with ChCM.

The PERC rule (pulmonary embolism rule-out criteria), which considers immobilization, malignancy, and history of deep vein thrombosis [14], is useful to rule out PE in non-Chagas disease patients when the patient’s score is zero. In the setting of ChCM, the PERC rule might be of some value to exclude the diagnosis of PE. Nevertheless, the problem is that in those patients with CHF present at the emergency department, the median age is 57 years [35]; this will yield a patient’s score equal to one, and consequently PE will not be excluded in a substantial number of patients.

The Wells system is another model used to predict PE in non-Chagas disease patients, which considers recent immobilization, malignancy, and history of deep vein thrombosis [13]. It might be useful especially in patients with ChCM with a low probability of PE but with one item of PERC score (for example, haemoptysis). In such patients, D-dimer testing might be of value before chest imaging.

The Geneva score [15] has also been validated to predict the diagnosis of PE in non-Chagas disease patients. It also considers immobilization, deep vein thrombosis, malignancy, and haemoptysis. However, heart rate on physical examination >75 beats per minute and age >65 years are important components of this model. In the context of ChCM, in patients who present with a median age of 57 years and a median heart rate of 71 beats per minute [35], it is conceivable that the Geneva score will not perform well for the diagnosis of PE.

Another score (the Vienna score) integrates sex, thrombosis site, and D-dimer, to estimate the probability of recurrent venous thromboembolism in patients with deep-vein thrombosis [36]. No correlation has yet been established between right-sided cardiac thrombosis and D-dimer serum levels in patients with ChCM. Therefore, whether the Vienna score can be useful to diagnose right-sided cardiac thrombosis in patients with ChCM remains to be determined. Collectively, the scores used to predict PE in non-Chagas disease will be of little value for patients with PE associated with ChCM

The high frequency of cardiac thrombosis and thromboembolism in patients with chronic Chagas disease has traditionally been considered to be the result of blood stasis and endocardial myocardial inflammation [5]. In fact, in patients with chronic Chagas disease, cruzipain (a molecule derived from *T. cruzi*), activates bradykinin receptors, thus enhancing cell invasion [37]. In addition, cruzipain also interacts with kininogens further facilitating cell invasion [38]. This can account, at least in part, for the extension of myocardial inflammation until the endocardium in patients with ChCM. Other mechanisms, such as proinflammatory cytokine overexpression [39], platelet hyper aggregation [37], platelet human polymorphism [40], *T. cruzi*-induced vascular endothelium damage [41], and high serum levels of prothrombotic variables [42] may also play a role in the pathogenesis of cardiac thrombosis in patients with ChCM.

This study has several limitations. The small sample size of the studies included in this investigation precludes a firm conclusion about the clinical aspects associated with PE in patients with ChCM, and the establishment of risk factors for PE in patients with this condition. An important limitation of this study is the lack of a Pulsed-Wave Tissue Doppler Imaging (PW-TDI) analysis of thrombus mobility. In non-Chagas disease patients with left ventricular thrombi, 17 out of 72 (23%) patients had systemic embolism. In that study, risk factors for systemic embolism were mobile thrombi with a free edge as well as mass peak antegrade velocity [43]. In the setting of chronic Chagas heart disease, no previous PW-TDI study has previously been carried out. Therefore, PW-TDI might be useful to refine the risk of PE in patients with chronic Chagas disease patients and right-sided cardiac thrombus.

Another limitation of this investigation is the lack of a detailed echocardiographic study of the right-sided heart in the papers included in the investigation. In fact, the dilatation of the inferior vena cava, the right cardiac diameters, the paradoxical displacement of the interventricular septum, the mean gradient of the tricuspid valve, the mean pressure of the pulmonary artery, and the diameter of the pulmonary artery, were not evaluated in the papers included in the study. The last limitation is that only one patient was reported to have a computed tomography angiography, which is crucial for the diagnosis of PE.

## 6. Conclusions

In summary, this review shows that the clinical diagnosis of PE in patients with ChCM is very difficult in the absence of a prediction score that performs well in patients with this condition. However, our review shows for the first time that in the presence of haemoptysis or worsening HF, abnormal ECG, or chest X-ray, the diagnosis of PE should be raised, and patients promptly referred to detailed Doppler Tissue Echocardiography, computed tomography angiography, and treated in a timely manner. This may be useful for doctors working in distant rural areas of Latin America. Nonetheless, further studies are urgently required to comprehensively guide the diagnosis of this lethal disorder.
